# CNPase, a 2′,3′-Cyclic-nucleotide 3′-phosphodiesterase, as a Therapeutic Target to Attenuate Cardiac Hypertrophy by Enhancing Mitochondrial Energy Production

**DOI:** 10.3390/ijms221910806

**Published:** 2021-10-06

**Authors:** Keai Sinn Tan, Dongfang Wang, Ziqiang Lu, Yihan Zhang, Sixu Li, Yue Lin, Wen Tan

**Affiliations:** 1 College of Pharmacy, Jinan University, Guangzhou 510632, China; tankeaisinn@jnu.edu.cn (K.S.T.); dfwang@jnu.edu.cn (D.W.); 2 Post-Doctoral Innovation Base, Jinan University Affiliation, Yuanzhi Health Technology Co., Ltd., Hengqin New District, Zhuhai, Guangdong 51900, China; luzq85@126.com (Z.L.); bettyscu@163.com (Y.Z.); lisx_96@163.com (S.L.); catbrighton@163.com (Y.L.); 3 Jeffrey Cheah School of Medicine and Health Sciences, Monash University Malaysia, Bandar Sunway 47500, Malaysia

**Keywords:** CNPase, heart failure animal model, zebrafish, CRISPR-Cas9, mitochondrial energy production

## Abstract

Heart failure is the end-stage of all cardiovascular diseases with a ~25% 5-year survival rate, and insufficient mitochondrial energy production to meet myocardial demand is the hallmark of heart failure. Mitochondrial components involved in the regulation of ATP production remain to be fully elucidated. Recently, roles of 2′,3′-cyclic nucleotide-3′-phosphodiesterase (CNPase) in the pathophysiological processes of heart diseases have emerged, implicated by evidence that mitochondrial CNPase proteins are associated with mitochondrial integrity under metabolic stress. In this study, a zebrafish heart failure model was established, by employing antisense morpholino oligonucleotides and the CRISPR-Cas9 gene-editing system, which recapitulates heart failure phenotypes including heart dysfunction, pericardial edema, ventricular enlargement, bradycardia, and premature death. The translational implications of CNPase in the pathophysiological process of heart failure were tested in a pressure overload-induced heart hypertrophy model, which was carried out in rats through transverse abdominal aorta constriction (TAAC). AAV9-mediated myocardial delivery of CNPase mitigated the hypertrophic response through the specific hydrolysis of 2′-3′-cyclic nucleotides, supported by the decrease of cardiac hypertrophy and fibrosis, the integrity of mitochondrial ultrastructure, and indicators of heart contractility in the AAV9-TAAC group. Finally, the biometrics of a mitochondrial respiration assay carried out on a Seahorse cellular energy analyzer demonstrated that CNPase protects mitochondrial respiration and ATP production from AngII-induced metabolic stress. In summary, this study provides mechanistic insights into CNPase-2′,3′-cyclic nucleotide metabolism that protects the heart from energy starvation and suggests novel therapeutic approaches to treat heart failure by targeting CNPase activity.

## 1. Introduction

Heart failure is the end-stage of cardiovascular disease and represents the top threat to public health worldwide. Despite accomplishments in therapeutics for heart failure, with a 5-year mortality rate of 75%, the prognostic results are far from satisfactory [1]. Cardiac pathological hypertrophy usually precedes the onset of heart failure and is characterized by enlarged cardiomyocytes, thickening, and stiff ventricular walls due to pressure overload, mutations of sarcomeric proteins, or infarction-induced cardiomyocyte loss [2]. The deficiency of ATP production plays a central role in the pathogenesis and progression of heart failure phenotypes [3]. At present, improving myocardial energy homeostasis with neuroendocrine systems and heart rate management has emerged as a core strategy for treating heart failure [4].

Genetic modification, pharmacological and surgical methods have been used in small animals to decipher the pathogenesis of heart failure and develop new therapies. Zebrafish are well characterized and widely used cardiovascular disease models based on genotypic and genotyping similarities. They are suitable for high-throughput chemical screening and gene manipulation due to their tiny size, relatively low maintenance cost, rapid development, accessibility for in vivo imaging, and the optical clarity of embryos [5]. Additionally, zebrafish embryos can survive without blood flow by oxygen diffusion, which makes them excellent models of heart failure [6]. Approximately 25% of mouse genes are embryonically lethal when knocked out [7], making it difficult to produce viable murine models for the study of heart failure.

Recently, the roles of 2′,3′-cyclic nucleotide-3′-phosphodiesterase (CNPase) in the pathophysiological processes of heart diseases have emerged, as indicated by our group targeting CNPase enzymatic activity, which might represent a novel therapeutic strategy for heart failure [8]. As an unconventional PDE, CNPase catalyzes 2′,3′-cyclic nucleotides as substrates [9], which are reported to impair mitochondrial integrity and accelerate the opening of the mitochondrial permeability transition pore (mPTP) [10]. As a mitochondrial protein, protein–protein interactions between the functional complexes I–V of the mitochondrial inner membrane and CNPase, leads to the assumption that CNPase might be involved in mitochondrial respiration and energy production [11]. Increasing shreds of evidence suggest that CNPase plays a role during ageing through substrate hydrolysis activity and protein–protein interactions to protect mitochondria from mPTP, and the antiaging activity of CNPase in heart failure and brain cognition was suggested by two reports [12,13]. However, CNPase deficiency is potentially embryo lethal, and premature deaths are quite common among CNPase −/− litters [14]. Thus, suitable genetically modified animal models, which could be developed as powerful tools to provide direct evidence of CNPase function in heart failure, are lacking for studying CNPase in cardiovascular diseases.

There is an unmet need to develop new therapies aiming to decrease the morbidity and mortality of heart failure. However, there is no direct evidence of whether CNPase deletion is physiologically deleterious and provokes proarrhythmic, structural changes in the myocardium that contribute to the pathologies of the heart failure syndrome. Herein, we investigated the translational implications of 2′,3′-cyclic nucleotide metabolism in the pathogenesis and progression of heart failure. By morpholino knockdown, a *cnpase* deficient zebrafish was established to explore the role of CNPase in the pathogenesis of cardiac diseases. To better understand the etiology of heart failure, we generated *cnpase* heterozygous knockout zebrafish strains. Finally, we assessed the therapeutic value of CNPase in a pressure-overload rat model, and the effects of CNPase on mitochondrial metabolism were investigated by the Seahorse cellular energy analyzer. Our results indicate that by targeting CNPase, mitochondrial function could be an effective strategy for pharmacological approaches to manage heart failure. 

## 2. Results

### 2.1. A Zebrafish Larvae Model of Spontaneous Dilated Cardiomyopathy Induced by Morpholino-Mediated Knockdown of CNPase

**Figure 1:**
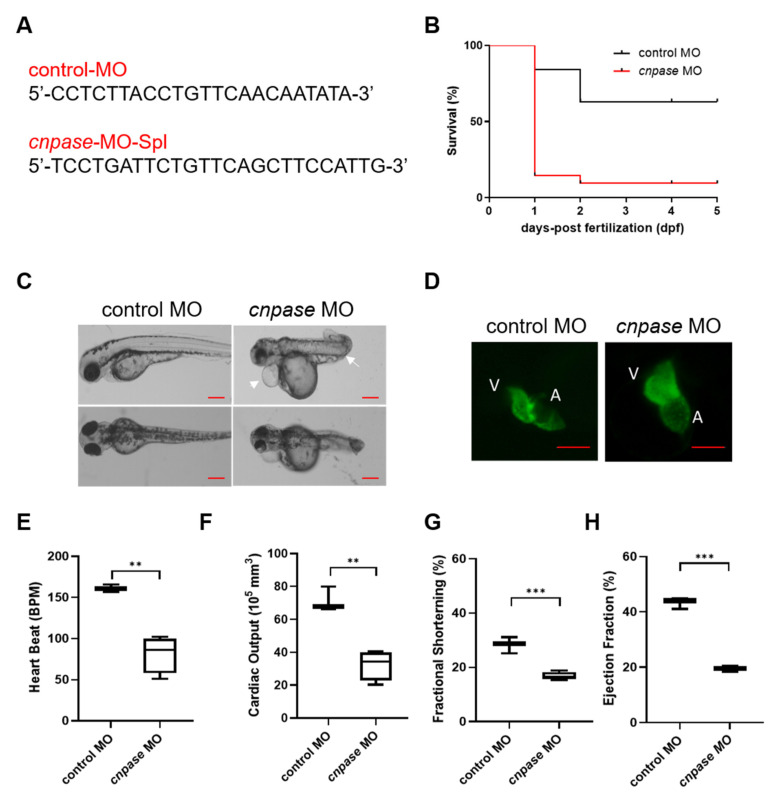
Morpholino-mediated cnpase knockdown results in a heart failure phenotype in zebrafish larvae. (**A**) The target sequences of control morpholino and morpholino targeting *cnpase*. (**B**) Effects of morpholino injection on the survival rate of zebrafish embryos over 120 hpf, *n* = 25~50. hpf, hours post-fertilization. (**C**) Phenotypes of the tg(*myl7:gfp*) zebrafish after *cnpase*-MO administration at 4 dpf. Arrows indicate the pericardial edema and malformation of body shape. dpf, days post-fertilization. Scale bar, 1000 µm. (**D**) cnpase-morpholino administration results in enlarged ventricular and atrium size. The images were captured by upright fluorescence microscope (Axio Imager A2, Zeiss). Scale bar, 1000 µm. (**E**–**H**) Cardiac function evaluation of zebrafish embryo after MO injection at 4 dpf, *n* = 25~50. Values are expressed as mean ± SD. Unpaired t-test, **, *p* < 0.005; ***, *p* < 0.001.

To explore the role of CNPase in cardiomyopathy development, a translation-blocking *cnpase* morpholino (MO) was designed (Figure 1A) and injected onto one-cell stage embryos. Kaplan–Meier analysis of the survival rate showed that roughly >85% of the *cnpase*-blocked larvae were deceased within three days post-fertilization, suggesting that the *cnpase* loss-of-function might lead to embryonic or neonatal death (Figure 1B). Notably, the *cnpase*-blocked larval zebrafish developed pericardial edema, atrium and ventricle hypertrophy, bradycardia with a reduced ejection fraction, fractional shortening, and cardiac output, which was highly similar to the pathophysiological state of human heart failure (Figure 1C–H). 

### 2.2. The Generation of a Zebrafish Cardiomyopathy Model by CRISPR-Cas9-Mediated CNPase Knockout 

**Figure 2:**
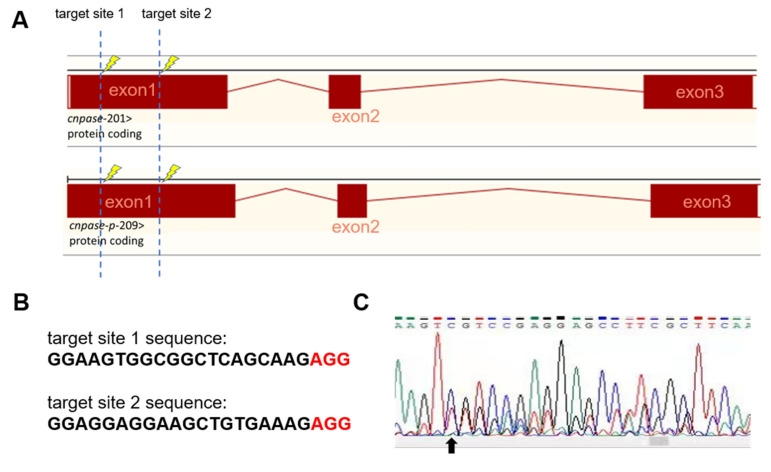

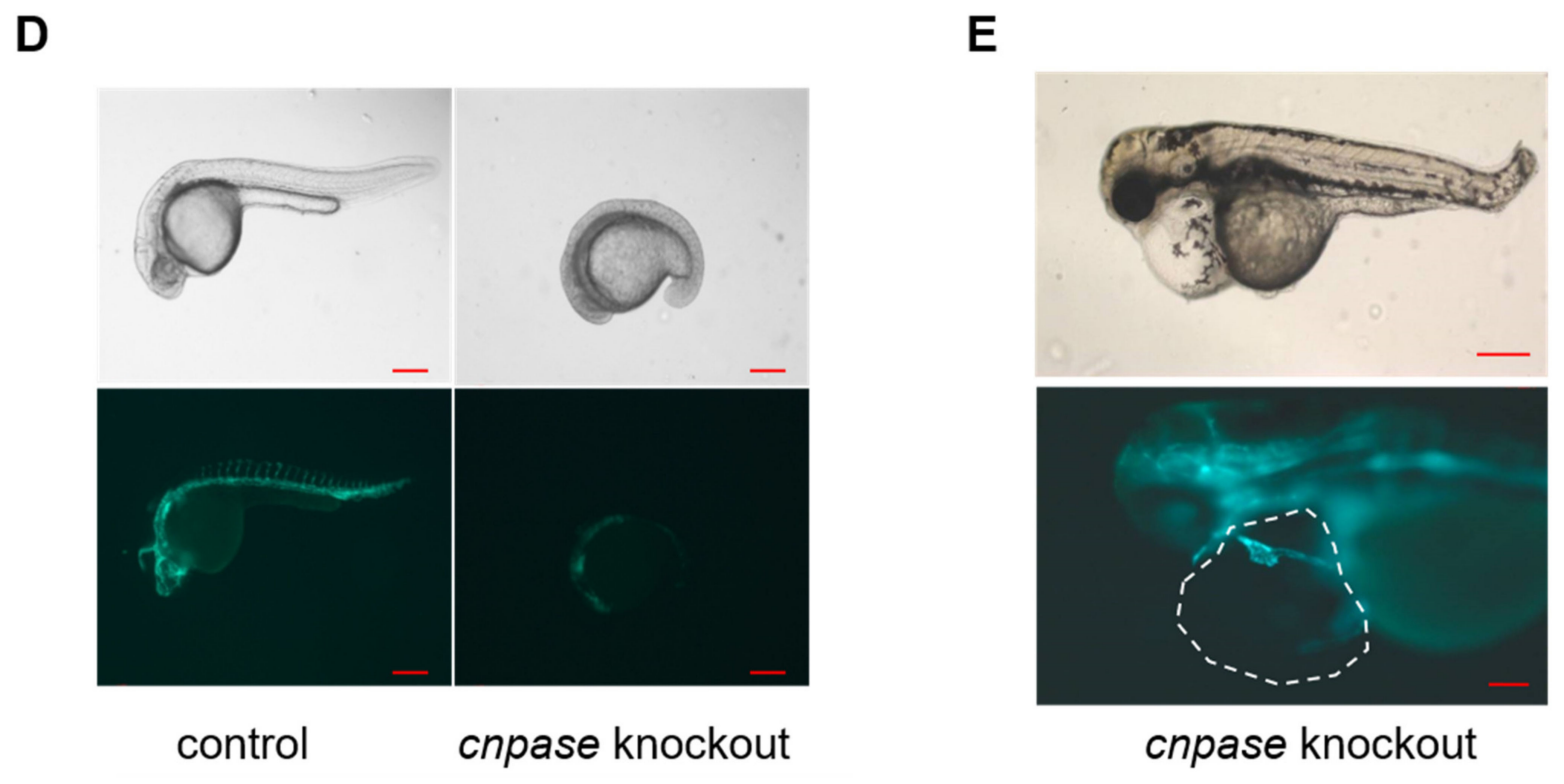
Generation of CNPase knockout zebrafish strain using the CRISPR-Cas9 editing system. (**A**) Schematic representation of the knockout strategy using CRISPR-Cas9 by targeting the first exon of *cnpase*. The cnpase-201, cnpase-209 encodes CNPase I (45 kDa) and CNPase II (47 kDa), respectively. (**B**) Sequences of the target site 1 and 2. “AGG” is the protospacer adjacent motif. (**C**) The genotyping of cnpase+/− zebrafish confirmed by tail-DNA PCR amplicons. The arrow indicates where the frame shift begins. (**D**,**E**) The *cnpase* deficient zebrafish exhibits developmental retardation, and cardiac failure phenotypes. Scale bar, 1000 µm.

To establish a stable cardiomyopathy animal model, we herein generated a *cnpase* knockout zebrafish strain on a pure AB Tg(*flk: egfp*) background using the CRISPR-Cas9 gene-editing system. CRISPR-Cas9 founders (F0) were generated in which the first exon of the zebrafish CNPase ortholog was targeted simultaneously by a pair of sgRNAs (Figure 2A). Adult founders were inbred to give birth to the offspring, in which indels were detected in the PCR amplicons of the region around the target site (Figure 2B,C). We found that frame-shift indels in the *cnpase* coding region could create mutant alleles in zebrafish in which it recapitulates MO phenotypes, excluding the possibility of MO-induced toxic and off-target effects (Figure 2C,D). Considering that homozygous mutations of CNPase cause embryo or neonatal lethality, the heterozygous *cnpase*+/− zebrafish strain was maintained for future study. 

### 2.3. AAV9-Mediated CNPase Myocardial Delivery Ameliorates Heart Dysfunction

**Figure 3:**
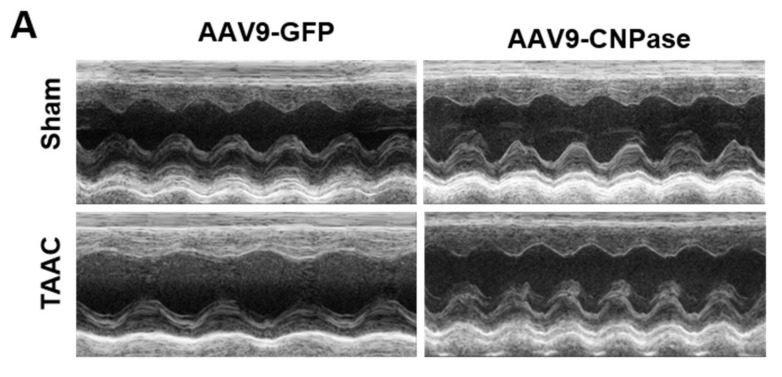

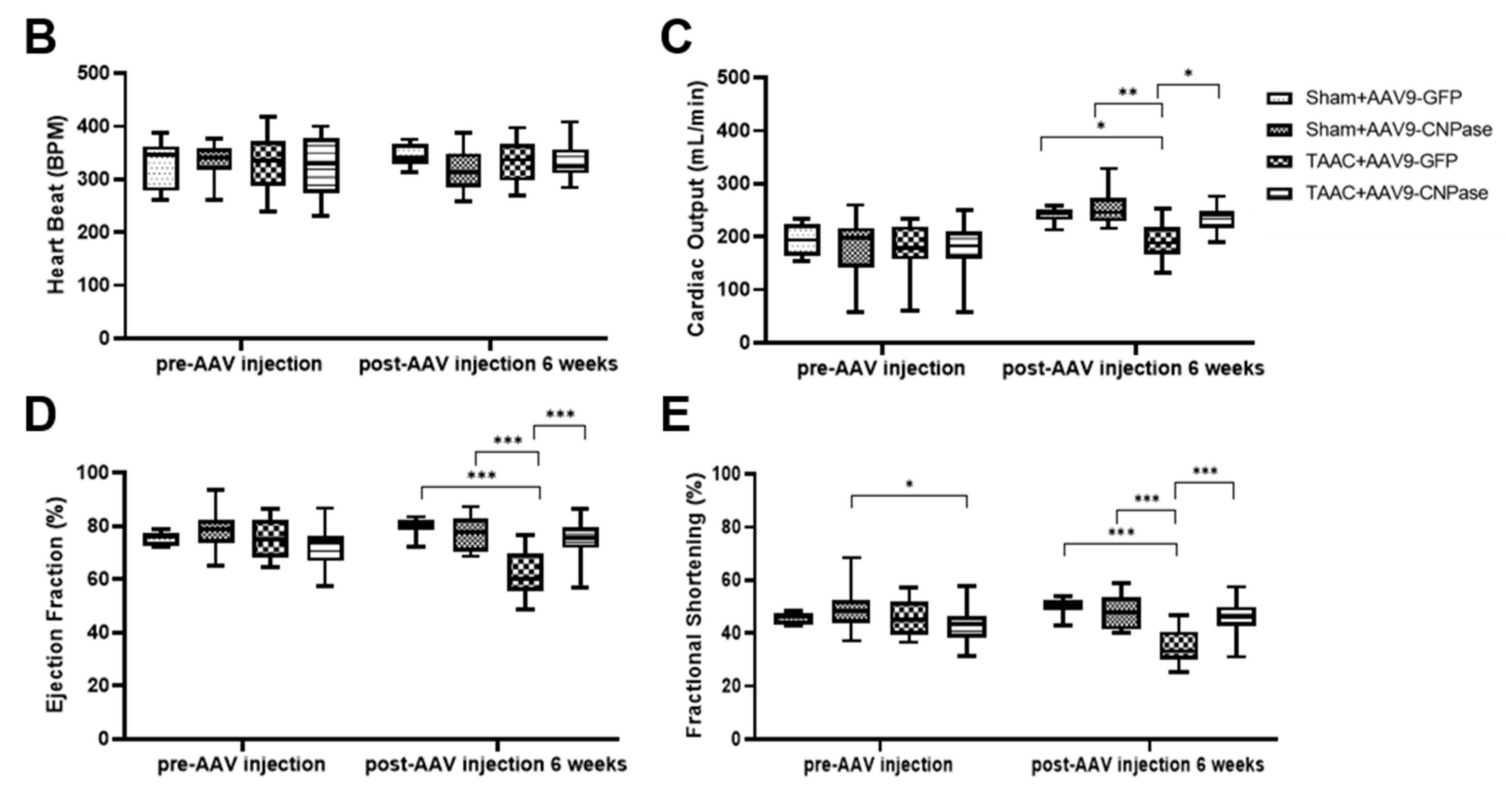
Myocardial CNPase delivery protects the heart from cardiac hypertrophy and dysfunction. (**A**) Representative echocardiography images of left ventricles. (**B**–**E**) The therapeutic effects of CNPase on heart rate, left ventricular ejection fraction, fraction shortening, cardiac output. The pressure overload-induced heart hypertrophy was carried out by transverse abdominal aorta constriction (TAAC) surgery. The AAV9-CNPase vectors (1 × 10^12^ genome copies) were delivered into the left ventricular myocardium guided by echocardiography. *n* = 8 in the sham group, *n* = 14–16 in the TAAC group. One-way ANOVA, Tukey’s multiple comparisons test, *, *p* < 0.05; **, *p* < 0.005; ***, *p* < 0.001.

Our group reported that targeting CNPase enzymatic activity might give rise to novel therapies for heart failure [8]. Thus, the therapeutic potential of CNPase as a new target in a translational cardiac hypertrophy animal model was explored. Heart hypertrophy was surgically induced by abdominal aorta constriction in Sprague-Dawley rats. Two weeks later, CNPase was introduced into the left ventricular myocardium through ultrasound-guided AAV9 delivery. Four weeks after the AAV9 delivery, the echocardiographic analysis revealed that the hypertrophic response was less pronounced in the AAV9-CNPase delivered group, supported by the decrease of left ventricular internal diameter in the vesicle-delivered group (Figure 3A). Further, the heart dysfunction had recovered upon the AAV9-mediated gene therapy, wherein the cardiac output (CO), left ventricular ejection fraction (EF), as well as fraction shortening (FS) were enhanced (Figure 3C–E). No effects of CNPase expression on heart rate were observed (Figure 3B).

### 2.4. AAV9-Mediated CNPase Myocardial Delivery Counteracts the Hypertrophic and Fibrosis Response

**Figure 4:**
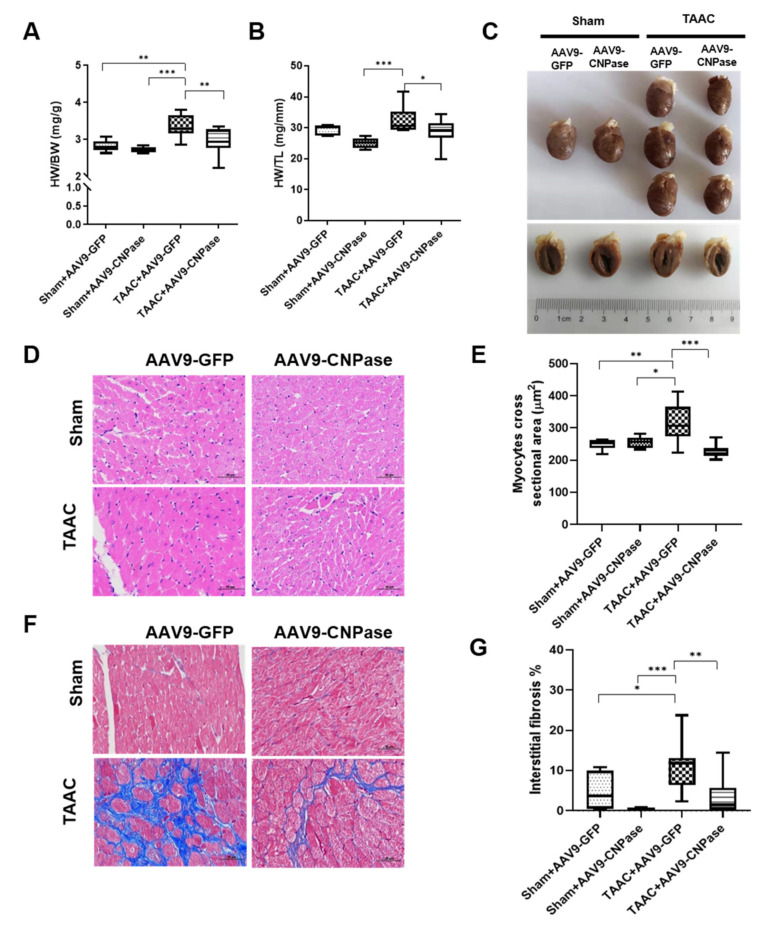

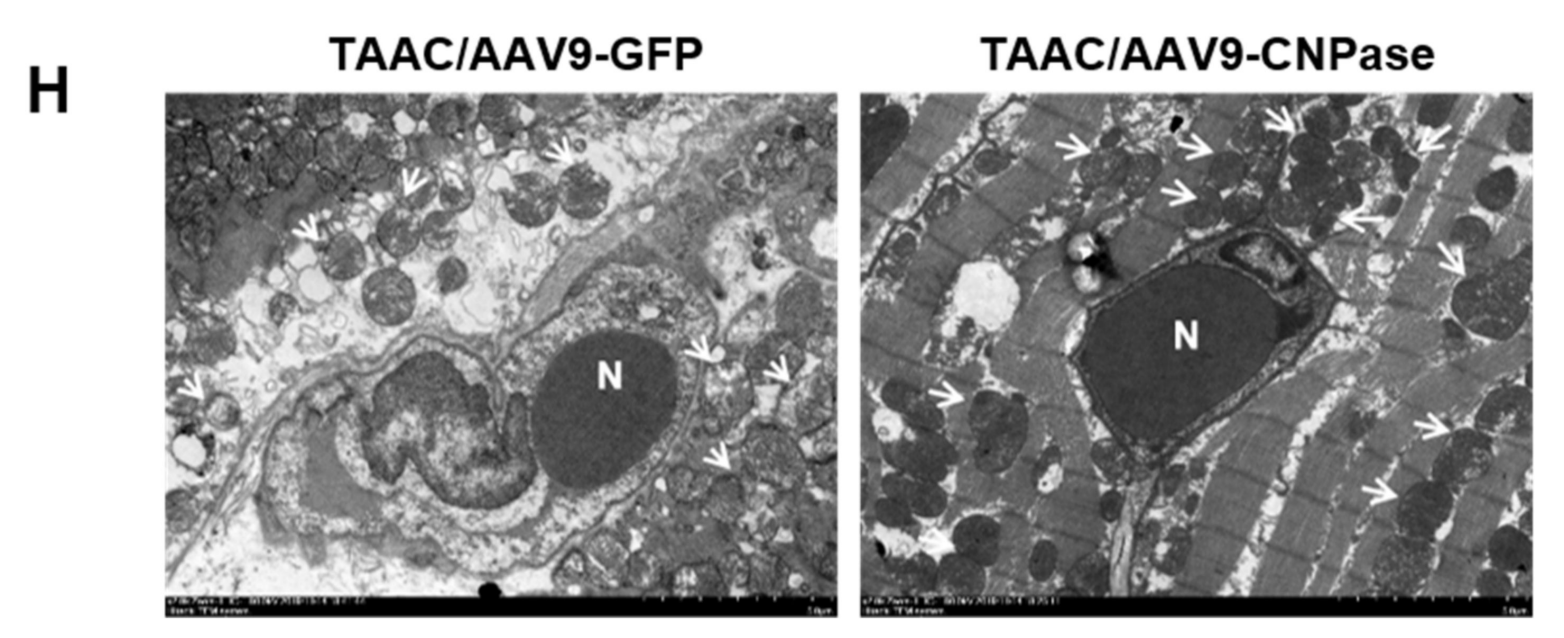
Myocardial CNPase delivery protects the heart from pressure overload-induced cardiomyopathy. (**A**,**B**) Effects of CNPase overexpression on the heart weight to body weight (HW/BW) ratio and the heart weight to tibia length (HW/TL) ratio. (**C**–**E**) Effects of CNPase overexpression on the heart hypertrophy and cardiomyocyte size. Representative images of heart cross sections by hematoxylin and eosin (H and E) staining and quantitative analysis. Original magnifications, D Â400. (**F**,**G**) Representative images of Masson’s Trichrome stained heart sections and quantification analysis. The pressure overload-induced heart hypertrophy was carried out by transverse abdominal aorta constriction (TAAC) surgery. (**H**) Representative transmission electron micrographs of mitochondria from the left ventricular myocardium in the AAV9-vehicle/TAAC and AAV9-CNPase/TAAC groups. Arrows indicating the representative mitochondria, scale bar, 5 µm. N, nucleus. The AAV9-CNPase vectors (1 × 10^12^ genome copies) were delivered into the left ventricular myocardium guided by echocardiography. *n* = 8 in the sham group, *n* = 14 in the TAAC-GFP group, *n* = 16 in the TAAC-AAV-CNPase group. One-way ANOVA, Tukey’s multiple comparisons test, *, *p* < 0.05; **, *p* < 0.005; ***, *p* < 0.001.

The hallmarks to indicate cardiac hypertrophy were the ratio for heart weight to body weight (HW/BW) and the ratio of heart weight to tibia length (HW/TL). Our data demonstrated that the hypertrophic myocardiopathy responses were attenuated upon the AAV9-CNPase delivery (Figure 4A,B). Figure 4C–E shows that the reduction of cardiomyocyte size could be responsible for the mitigation of cardiac hypertrophy in the AAV9-CNPase/TAAC group. The left ventricular section in the pressure overload-induced group was characterized by an excessive accumulation and a disrupted composition of extracellular matrix proteins, wherein the AAV9-CNPase administration trimmed the fibrosis response (Figure 4F,G). Taken together, our findings reveal that in the pressure overload-induced preclinical model, CNPase expression mitigates cardiac inflammation, hypertrophy, and failure in response to pressure overload, suggesting CNPase is playing the role of linking inflammation and hypertrophy in heart.

Compromised mitochondrial respiration is a hallmark of cardiac dysfunction. To investigate mitochondrial ultrastructure, transmission electron microscopic analysis demonstrated that myocardial fibers were beginning to lose cross striations, damaged mitochondria were associated with cristae lysis, and abnormal internal membrane whorls were present in the pressure-overload model group (Figure 4H). In comparison, the cardiac fibromuscular and mitochondrial ultrastructure characteristics were well preserved in the AAV9-CNPase group, despite the presence of pressure overload (Figure 4H).

### 2.5. Computational Analysis of the Dynamic Interaction between CNPase and Cyclic Nucleotide Isomers

**Figure 5:**
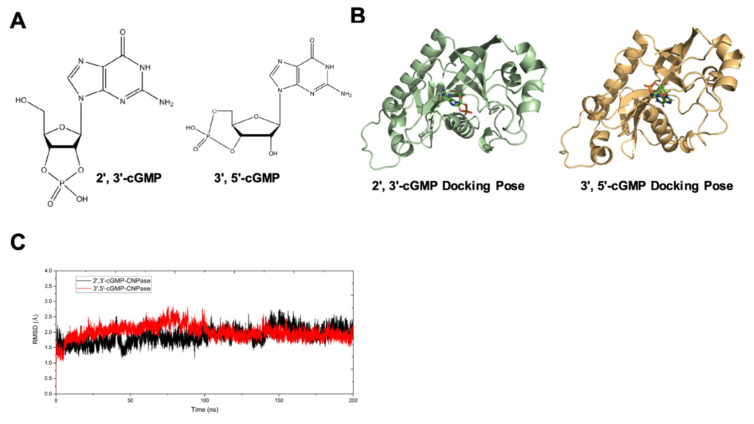
In silico simulation of the dynamic interaction of CNPase with 2′,3′-cGMP and 3′,5′-cGMP. (**A**) The chemical structures of 2′,3′-cGMP and 3′,5′-cGMP. (**B**) The docking complex of CNPase with 2′,3′-cGMP or 3′,5′-cGMP. (**C**) The time course of the root-mean-square deviation (RMSD) of the 2′,3′-cGMP-CNPase (black) and 3′,5′-cGMP-CNPase (red) complex system in a simulation of 200 ns.

The interaction of 2′, 3′-cGMP and CNPase was demonstrated in several studies [8]; however, structural comparisons of 3′, 5′-cGMP binding with CNPase were lacking. To explain the binding affinity and specificity of cyclic nucleotide isomers with CNPase, computational analysis of molecular docking, molecular dynamics (MD) simulations, and binding free energy calculations were carried out as previously reported [8]. Molecular structures of 2′,3′-cGMP and 3′,5′-cGMP were demonstrated (Figure 5A). Considering the binding free energy, and docking poses with the highest scores, the conformation and orientation of CNPase-cyclic nucleotide-binding complexes were determined (Figure 5B). 

To analyze the composition’s stability and the conformation changes of 2′,3′-cGMP and 3′,5′-cGMP with CNPase, the root-mean-square deviation (RMSD) was determined according to previously reported methods [8,15]. After a 200 ns MD simulation, the complex structure reached the equilibrium state (Figure 5C). However, the overall structural fluctuations of the 3′,5′-cGMP complex (red line) were larger than those of the 2′,3′-cGMP complex. In contrast, no apparent molecular distortion in the 2′,3′-cGMP–CNPase complex was observed during the simulations, further confirming that, compared to 3′,5′-cGMP, the 2′,3′-cyclic nucleotides are favorable substrates of CNPase (Figure 5C). The binding energy calculations reveal that the binding capacity of CNPase with 2′,3′-cGMP is favorable, compared to that of CNPase with 3′,5′-cGMP (Table 1). Since free energy barriers determine the folding and binding of CNPase with substrates, it could be speculated that the molecular conformation of CNPase is more accessible to 2′,3′-cyclic nucleotides, rather than 3′,5′-cyclic nucleotides.

### 2.6. CNPase Overexpression Improves Mitochondrial Respiration Function in Cardiac Hypertrophy

**Figure 6:**
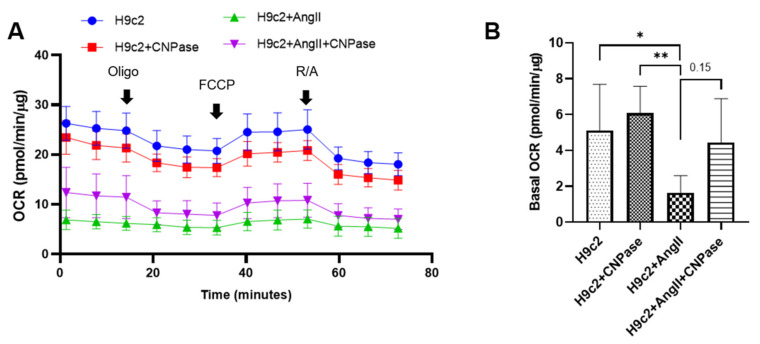

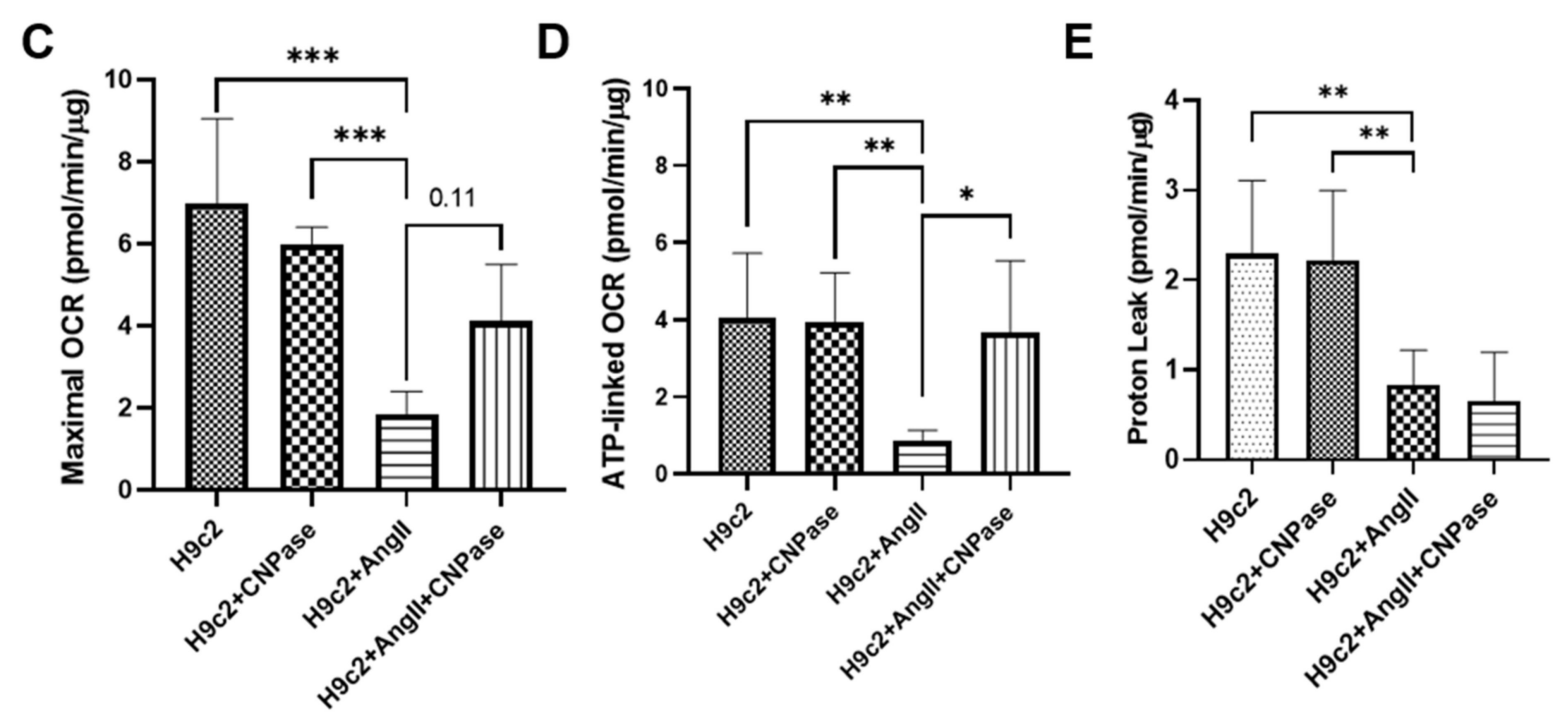
CNPase expression is essential for mitochondrial integrity, respiration capacity, and ATP production. (**A**) The general scheme of the mitochondrial stress test. The mitoStress assay was carried out on a Seahorse XFp extracellular flux analyzer (Agilent, Santa Clara, CA, USA) to directly measure the oxygen consumption rate (OCR) of cells. H9C2 cells were plated at a density of 2000 cells/well onto a 96-well seahorse microplate and transfected with pcDNA3.1-HA or pcDNA3.1-HA-CNPase vectors. For the mitoStress assay, programmable injection of selective inhibitors, oligomycin (2.5 µM), FCCP (1 µM), and rotenone/antimycin (2.5 µM) was performed. The Seahorse data shown are acquired from 8–12 independent replicates per experiment. Oligo, Oligomycin; FCCP, Potent uncoupler of mitochondrial oxidative phosphorylation; R/A, Rotenone/Antimycin. (**B**–**E**) Effects of CNPase overexpression on the basal, maximal and spare mitochondrial respiration, ATP production, and proton leak. One-way ANOVA, Tukey’s multiple comparisons test, *, *p* < 0.05; **, *p* < 0.005; ***, *p* < 0.001.

As the most energy-demanding organ, the heart relies on mitochondria as the powerhouse to produce ATP. The mitochondrial bioenergetics were profiled by the mitoStress assay using a Seahorse XFe96 cellular energy analyzer (Figure 6A). Our data demonstrated that the respiratory capacity was decreased upon AngII-induced hypertrophic stress. In contrast, the mitochondrial bioenergetics were well preserved in CNPase-overexpressed cells, as suggested by the basal, maximal, and reserved mitochondrial oxygen consumption rate (OCR) (Figure 6B–E). Notably, there was an increase of ATP-linked OCR in CNPase-overexpressed cells compared to the empty vector-transfected cells (Figure 6D). 

## 3. Discussion

The mechanism underlying mitochondrial energy production leading to heart failure is not fully elucidated. We found that zebrafish larvae with morpholino-mediated *cnpase* silencing develop a heart failure phenotype, featuring increased ventricular size, impaired contractility and relaxation, edema, and cardiac arrhythmia. Further, a zebrafish model with heart failure was developed using CRISPR-Cas9-mediated genetic manipulation. This animal model could be widely used to define the role of CNPase in mitochondrial energy production during heart failure. Finally, AAV9-mediated delivery of CNPase provides mechanistic insights into CNPase-2′,3′-cyclic nucleotide metabolism that protects the heart from energy starvation, and suggests novel therapeutic approaches to treat heart failure.

So far, little is known about the pharmacology and implicated application of the CNPase-2′,3′-cyclic nucleotide metabolic system. In contrast, therapeutic approaches based on the 3′,5′-cGMP/cAMP system, which give rise to drugs that block specific phosphodiesterases (PDEs), are still attracting great interest in the scientific research community, clinical and pharmaceutical industries [16]. However, phosphodiesterase-5 inhibition is not beneficial for heart failure patients with a preserved ejection fraction [17]. So far, no PDE inhibitors have been approved for chronic heart failure, except PDE5 inhibitors that were approved for treating pulmonary arterial hypertension. Nevertheless, clinical trials and practice are trying to identify new clinical uses of PDE5 inhibitors for treating heart failure with pulmonary vascular resistance, pulmonary hypertension secondary to left ventricular HF, and transplantation-related advanced heart failure. Thus, searching for novel pathophysiological mechanisms and therapies is an urgent need. Recent evidence suggests that CNPase represents a novel target for maintaining mitochondrial respiration by preventing mitochondrial calcium overload and mPTP via the hydrolysis of 2′,3′-cyclic nucleotides [10]. Reports indicate a decline of mitochondrial CNPase enzymatic activity in the pathophysiological state of heart failure, due to the hyperacetylation or release of CNPase proteins from mitochondria [8,17].

Antisense morpholino and CRISPR-Cas9-mediated *cnpase* gene-editing systems were employed in this study to develop a heart failure zebrafish model (Figure 1 and Figure 2). Zebrafish deficient in *cnpase* develop visibly slow heart rates, pericardial edema, and a distorted cardiac structure (Figure 1C). The survival rate of zebrafish embryos after MO-mediated *cnpase* knockdown was lower than that of control MO at a high dose (Figure 1B). The heart rate, cardiac output, fractional shortening, and ejection fraction were severely decreased in *cnpase* MO larvae, suggesting that the zebrafish model with heart failure was successfully established (Figure 1). Notable pericardial edema suggests the involvement of CNPase in inflammation activation, that in concert with CNPase expression, is associated with microglial activation and controls the expression of inflammatory cytokines [18]. This study sheds light on CNPase-2′,3′-cyclic nucleotide metabolism as a new pathophysiological mechanism responsible for cardiac remodeling, based on which new therapy approaches for heart failure could be developed.

Mismatched energy production and demand is the hallmark of heart failure. In this sense, CNPase represents as novel “energy” therapy target for heart failure beyond the traditional neurohumoral and hemodynamic modulation approaches. The CNPase-2′,3′-cyclic nucleotide system is present in the circulating system, and cardiomyopathy could increase the formation of 2′,3′-cyclic nucleotides through mRNA degradation [19]. AAV9-mediated CNPase myocardial expression alleviated the pressure overload-induced hypertrophic response and improved heart function (Figure 3 and Figure 4), possibly by CNPase metabolizing 2′,3′-cyclic nucleotides into adenosine to exert beneficial physiological effects [19,20]. Our study also explains the binding affinity of CNPase proteins with cyclic nucleotide positional isomers (Figure 5), concluding that CNPase does not interfere with PDE 3′-5′-cyclic nucleotide pathways. The cellular mitochondrial analysis indicated that CNPase protects the hypertrophic heart from energy depletion by preserving the mitochondrial respiration and ATP production capacity (Figure 6). The presence of 2′-3′-cyclic nucleotides have been identified in immune cells [21]; however, pieces of evidence of 2′-3′-cyclic nucleotides triggering inflammation are still lacking. As elucidated by Zhang etc., 2′,3′-cGMP was identified as a potent endogenous ligand binding to TLR7 family proteins [22], which recognize virus ssRNAs to activate the NF-κB pathway and produce inflammatory cytokines. TLR7 activation contributes to systemic inflammation, myocarditis and dilated cardiomyopathy [23]. As an agonist of TLR7, abnormal accumulation of 2′,3′-cGMP could contribute to the inflammatory and fibrotic responses in the pathophysiology of heart failure. This might give clues how the ability of CNPase to degrade 2′,3′-cGMP versus 3′,5′-cGMP is advantageous in heart failure. Taken together, these findings suggest myocardial CNPase delivery plays a therapeutic role modulating cardiac hypertrophy, energetic dysfunction, arrhythmia, extracellular matrix remodeling, and inflammation.

Meanwhile, a study of CNPase in the renal system suggests that our understanding of CNPase is still preliminary. In contrast to this study, CNPase deficiency alleviated the severity of acute kidney failure during ischemia–reperfusion, and suggested that CNPase deficiency protects the kidney from oxidation stress by inducing autophagy and removing damaged mitochondria [24]. Other than 2′-3′-cyclic nucleotide PDE activity, CNPase proteins could promote lipid raft distribution, tRNA maturation, microtubule assembly, and a change of mRNA stability and translation [25].

There are a few drawbacks in this study, and further investigation is required to better elucidate the function and underlying mechanism of CNPase. First, a rescue experiment is needed to investigate whether the reintroduction of *cnpase* proteins could rescue the pathophysiological phenotype, although a second method using the CRISPR-Cas9 system validated the genotyping and phenotyping of *cnpase*. Second, in vivo energy expenditure and metabolic biomarker metrics during CNPase therapy are lacking, and the effects of CNPase expression needs to be fully determined. Finally, the molecular role and underlying mechanism of CNPase are not well characterized in this study. So far, a report reveals that CNPase regulates the epithelial–mesenchymal transition of lens epithelial cells through the notch signaling pathway [26].

In summary, a zebrafish model with heart failure was developed targeting *cnpase* by using morpholino and gene-editing systems. The therapeutic potential of CNPase was validated in a pressure overload-induced cardiac hypertrophy model by the myocardial introduction of AAV9-CNPase, which ameliorated cardiac hypertrophy, fibrosis, and dysfunction through increasing cardiac energy production. 

## 4. Materials and Methods

### 4.1. Zebrafish Husbandry, Microinjection, Fluorescence Analysis of Heart Function

AB wild-type and Tg(myl7:GFP) transgenic strains were purchased from the Institute of Hydrobiology, Chinese Academy of Sciences and maintained in Zebrafish Aquatic Housing Systems (Shanghai Haisheng Biotech, Shanghai, China) at 28 °C, pH 7.0–8.0, 0.25% salinity, and a light-dark cycle 14L:10D. 

Morpholino antisense oligos targeting zebrafish CNPase mRNA were designed and synthesized (Gene Tools, LLC, Eugene, OR, USA). Embryos were injected (1 ng) at the one-cell stage by using an ASI pressure injection apparatus (Applied Scientific Instrumentation, Eugene, OR, USA). MO-injected embryos were grown in a 10-cm dish containing Holtfreter’s solution at 28 °C. The survival curve was plotted using a Kaplan–Meier Survival Curve.

Heart function was analyzed with fluorescence microscopy (ZEISS Axio Observer for Life Science Research, Jena, Germany). Zebrafish larvae (4 dpf) were anesthetized in 0.03% tricaine and mounted on 3% methylcellulose. Videos were recorded (15 s, repeated three times, 488 nm excitation wavelength) and processed using Adobe Bridge CC 2020 and Adobe Premiere Pro V8.1.0.7 software. The width and length of the ventricules were measured at the end of diastole and systole to obtain cardiac dimensions, based on which the heart function parameters were obtained [27].

### 4.2. Animal Husbandry, Abdominal Aortic Constriction Surgery, 2D Ultrasound Analysis of Heart Function

Sprague-Dawley male rats were bought from the Animal Center of Jinan Pengyue Laboratory Animal Breeding Co., Ltd. (Jinan, Shandong, China). Rats were housed with free access to water and standard chow, at room temperature 25 °C, relative humidity 40%–60%, and a standard 12 h light–dark cycle (light on/off at 7 a.m./7 p.m.). All animal studies were conducted according to the Guidance Suggestions for the Care and Use of Laboratory Animals, formulated by the Ministry of Science and Technology of China. The present study was approved by the Jinan University Animal Care and Use Committee (Ethics code: SCXK-20190306) in March 2019.

Transverse abdominal aortic constriction (TAAC) surgery was performed to induce cardiac pressure overload, which progressively led to myocardial hypertrophy and dysfunction. Briefly, animals were anesthetized with sodium pentobarbital (45 mg/kg) by intraperitoneal (i.p.) injection. The abdominal cavity was opened by performing a medial incision of the abdominal wall to expose the internal organs. After locating the abdominal aorta, the vessel sheath was dissected to expose the abdominal aorta. A 0.7 mm OD blunted needle was tied in place with a 3.0 mm suture, after which the needle was withdrawn, leaving the ligature in place. A diminution in the pulse amplitude and the paleness of the left kidney demonstrated the success of the ligature. Sham-operated rats were subjected to the same surgical procedure except that the aorta was not constricted. After 2 weeks, heart function was analyzed using ultrasound to evaluate the cardiac hypertrophic response. Guided by contrast echocardiography, the AAV9 (1 × 10^12^ genome copies) was delivered into the anterior and posterior left ventricular walls by a 30 G 0.5-inch needle. The total volume was 100 µL, and the injection was given five times to different positions. Transthoracic echocardiographic measurement was carried out at 4, 6, and 8 weeks after the surgery.

Cardiovascular phenotyping for cardiac structure and function was assessed using the Vevo 3100 Preclinical Imaging System (FUJIFILM, VisualSonics, Toronto, OT, Canada), after rats were anesthetized with 3%, 600 mL/min isoflurane with a Rodent Anesthesia Machine (GasAnesthesia, Coral Springs, FL, USA). The cardiac structure was visualized by photoacoustic contrast agents with a Vevo Optical Fibers probe (6 MHz~19 MHz), based on which, Vevo Lab software was used to analyze the left ventricular ejection fraction, fraction shortening, and cardiac stroke volume. The echocardiographic measurement was taken for at least three consecutive cardiac cycles.

### 4.3. Production, Purification, and Titration of AAV9-CNPase, and Ultrasound-Guided Left Ventricular Injection

Rat full-length CNPase was subcloned into the AAV transfer plasmid with the myocardial-specific cTnT promoter. The AAV was packaged and purified through iodixanol gradient purification to remove empty capsids (Vigene Biosciences, Jinan, Shangdong, China). Briefly, the triple plasmids involving AAV-CNPase, AAV9 Rep-Cap, and helper plasmids were introduced into AAV-293 cells, then the viral particles were harvested by freeze/thaw of the cell pellet to release the AAV virus. The AAV was stored at –80 °C and titrated by the qRT-PCR method to detect viral ITR genes. The AAV9-GFP virus was packaged as for the AAV9-CNPase virus except that the AAV9-GFP transfer plasmid was used during the co-transfection.

### 4.4. The CRISPR-Cas9 System-Mediated Genome Editing to Develop the CNPase Knockout Zebrafish Strain

The 20-nt sequence pair (GGAAGTGGCGGCTCAGCAAGAGG, GGAGGAGGAAGCTGTGAAAGAGG) targeting the first exon of CNPase was designed by the MIT CRISPR Tool. The gRNAs were prepared by in vitro transcription (mMESSAGE mMACHINE T3 kit, AM1348, Life Technologies, Carlsbad, CA, USA) using annealed oligos as the template, then RNA was purified with isopropanol/sodium acetate and quantified with Nanodrop2000. The SpCas9 protein was ordered from Novoprotein Scientific Inc., Shanghai, China. 

To create mutant alleles in zebrafish, the CRISPR mRNA/gRNA mix (1 nL, 25 pg sgRNAs, and 300 pg Cas9) was injected into one-cell stage Tg(flk: EGFP) transgenic zebrafish embryos. The cardiac phenotype was observed in the embryonic stage at 72 hpf. The homozygous mutant fish were screened from the offspring of the founder fish using PCR, subcloning, and sequencing. 

### 4.5. Histological and Morphological Analyses

Rat hearts were harvested and arrested in diastole with a 10% KCl solution. Then, the hearts were washed and fixed in 4% formalin, dehydrated through a graded series of alcohols, and embedded in paraffin wax. Subsequently, the left ventricles were cut into 5 µm thick sections, deparaffinized, and rehydrated before hematoxylin and eosin (H and E) staining and Masson’s Trichrome staining. The cardiomyocyte cross-sectional area and fibrosis area fraction were quantified by Image-Pro Plus v.6.0 (Media Cybernetics, Silver Spring, MD, USA).

Left ventricular tissues were dissected into small cubic pieces (≤1 mm^3^) and fixed with 2.5% glutaraldehyde, pH 7.4 for 3 h. After being washed in 0.1 M phosphate buffer three times, the tissues were fixed in 1% osmium tetroxide. Then samples were dehydrated by graded ethanol with the last dehydration procedure in 90% acetone. All above procedures were conducted at 4 °C. After being embedded in Epon Araldite and fixed, ultrathin sections (60–80 nm) were sliced using a Leica EM UC7 ultramicrotome (Leica, Wetzlar, Germany) and stained with 3% uranyl acetate and lead citrate. Images were captured with a HT7700 transmission electron microscope (Hitachi, Tokyo, Japan).

### 4.6. Cell Culture, Transient Transfection, and the Mitochondrial Biogenetics Analysis Using a MitoStress Assay

The rat embryonic cardiomyocyte-derived cell line H9c2 was purchased from the National Collection of Authenticated Cell Cultures (Chinese Academy of Sciences, Shanghai, China). H9c2 cells were cultured in DMEM medium supplemented with 10% fetal bovine serum and 0.1% penicillin-streptomycin in a humidified incubator with 5% CO_2_ at 37 °C. The in vitro hypertrophic model was established by AngII (1 µM) and serum starvation for 24 h. The HA-tagged CNPase plasmids were constructed and transiently transfected using Lipofectamine 2000 as described previously [8]. 

The Seahorse XF Cell Mito Stress assay was carried out on a Seahorse XFe96 Analyzer to profile mitochondrial biogenetics kinetics according to the manufacturer’s instructions. The basal respiration rate was measured in an XF assay medium supplemented with 1 mmol/L sodium pyruvate, 2 mmol/L l-glutamine, and 10 mmol/L glucose. Following that, the programmable injection of oligomycin (1 µmol/L), carbonyl cyanide (trifluoromethoxy) phenylhydrazone (FCCP) (1.5 µmol/L) and rotenone/antimycin A (R/A) (1 µmol/L) was performed in sequence from ports A, B and C, respectively. The assay was recorded for 150 min, and the OCR readings were normalized with the protein concentration. 

### 4.7. In Silico Simulation

The structure of human wild-type CNPase (PDB 1WOJ) was downloaded from the RCSD Protein Data Bank. Missing residues were modelled on UCSF Chimera 1.13.1 using the in-built Modeller tool. Molecular dynamic (MD) simulations of CNPase were carried out in Gromacs 2019.1 on an in-house Linux-based desktop computer. The protonation states of all protein residues were fixed at pH 7.0 and the simulations were carried out under a Gromos 54a8 force field. A water cubic box, extended 15 Å from the protein, was filled with TIP3 water molecules. The cutoff for short range interactions was set to 10 Å. A PME method was used for long-range electrostatic interactions. Periodic Boundary Conditions (PBS) were applied in all directions. Cl− anions were added to make the system neutral. Energy minimization was performed using the steepest descent algorithm with an energy convergence cutoff of 10.0 kJ/mol. Temperature and pressure equilibration was performed for 0.5-ns position-restrained MD simulations. Three productive MD simulations per structure were performed for 100-ns with a time step of 2 fs at a constant 1 atm pressure and 310 K temperature. Temperature was controlled using the modified Beredsen thermostat, and pressure coupling was performed using the Berendsen barostat. The three MD simulations were concatenate and calculated using built-in utilities installed on Gromacs [8].

### 4.8. Statistical Analysis

The results are expressed as the mean ± SD. A two-tailed Student’s *t*-test was used to assess the differences between two experimental groups, while a one-way ANOVA with post-hoc Tukey’s test was used for multiple group comparisons. A value of *p* < 0.05 was considered statistically significant.

## Figures and Tables

**Table 1:** Binding free energies (kcal/mol) for CNPase with 2′,3′-cGMP and 3′,5′-cGMP.

System	ΔEVDW	ΔEELE	ΔGGB	ΔGSURF	ΔGTOT	TΔS	ΔGBinding
2′,3′-cGMP	−41.62 ± 2.52	−138.93 ± 10.81	145.41 ± 8.85	−4.024 ± 0.12	−39.16 ± 4.09	−13.98 ± 4.53	−25.18 ± 6.10
3′,5′-cGMP	−23.61 ± 7.85	−151.90 ± 51.86	165.92 ± 47.92	−2.76 ± 0.80	−12.35 ± 6.060	−21.10 ± 5.83	8.76 ± 8.41

ΔEVDW, van der Waals energy; ΔEELE, electrostatic interaction energies; ΔGGB, polar solvation free energies; ΔGSURF, surface energies; ΔGTOT, total energies; TΔS, conformational entropy; ΔGBinding, binding energies.

## Data Availability

The data presented in this study are available in the article.
